# Evaluation of Diagnostic and Therapeutic Approaches for Suspected Influenza A(H1N1)pdm09 Infection, 2009–2010

**DOI:** 10.3201/eid1809.111564

**Published:** 2012-09

**Authors:** Vini Vijayan, Jennie Jing, Kenneth M. Zangwill

**Affiliations:** Los Angeles Biomedical Research Institute at Harbor–UCLA Medical Center, Torrance, California, USA

## Abstract

Variations between practice and national recommendations could inform clinical education in future influenza seasons.

## Abstract 2

Keywords: influenza; oseltamivir; H1N1, influenza A(H1N1)pdm09, viruses

## Medscape CME Activity

Medscape, LLC is pleased to provide online continuing medical education (CME) for this journal article, allowing clinicians the opportunity to earn CME credit. 

This activity has been planned and implemented in accordance with the Essential Areas and policies of the Accreditation Council for Continuing Medical Education through the joint sponsorship of Medscape, LLC and Emerging Infectious Diseases. Medscape, LLC is accredited by the ACCME to provide continuing medical education for physicians.

Medscape, LLC designates this Journal-based CME activity for a maximum of 1 *AMA PRA Category 1 Credit(s)*^TM^. Physicians should claim only the credit commensurate with the extent of their participation in the activity.

All other clinicians completing this activity will be issued a certificate of participation. To participate in this journal CME activity: (1) review the learning objectives and author disclosures; (2) study the education content; (3) take the post-test with a 70% minimum passing score and complete the evaluation at www.medscape.org/journal/eid; (4) view/print certificate.

**Release date: August 21, 2012; Expiration date: August 21, 2013**

## Learning Objectives

Upon completion of this activity, participants will be able to:

- Analyze the use of diagnostic testing in cases of influenza-like illness
- Evaluate the use of antiviral medications for outpatient cases of influenza-like illness
- Evaluate the use of antiviral medications for inpatient cases of influenza-like illness
- Assess the care of patients with influenza-like illness and lower respiratory tract infections

## CME Editor

**Carol E. Snarey, MA, **Technical Writer/Editor, *Emerging Infectious Diseases. Disclosure: Carol E. Snarey, MA, has disclosed no relevant financial relationships.*

## CME Author

**Charles P. Vega, MD, **Health Sciences Clinical Professor; Residency Director, Department of Family Medicine, University of California, Irvine. *Disclosure: Charles P. Vega, MD, has disclosed no relevant financial relationships.*

## Authors

*Disclosures:* ***Vini Vijayan, MD;*** *and* ***Jennie Jing, MS,*** *have disclosed no relevant financial relationships.* ***Kenneth Zangwill, MD,*** *has disclosed the following relevant financial relationships: served as an advisor or consultant for Merck & Co. Inc.; received grants for clinical research from Novartis.*

## References

[1] World Health Organization. New influenza A (H1N1) virus: global epidemiological situation June 2009. Wkly Epidemiol Rec. 2009;84:249–57. 19537358

[2] Novel Swine-Origin Influenza A (H1N1) Virus Investigation Team; Dawood FS, Jain S, Finelli L, Shaw MW, Lindstrom S, et al. Emergence of a novel swine-origin influenza A (H1N1) virus in humans. N Engl J Med. 2009;360:2605–15. 10.1056/NEJMoa0903810 19423869

[3] Centers for Disease Control and Prevention. Hospitalized patients with novel influenza A (H1N1) virus infection—California, April–May, 2009. MMWR Morb Mortal Wkly Rep. 2009;58:536–41. 19478723

[4] Centers for Disease Control and Prevention. Flu activity and surveillance, 2010 −2011 [cited 2011 Jun 5]. http://www.cdc.gov/flu/weekly/.

[5] Centers for Disease Control and Prevention. Interim guidance for the detection of novel influenza A virus using rapid influenza diagnostic tests [cited 2010 Dec 4]. http://www.cdc.gov/h1n1flu/guidance/rapid_testing.htm

[6] Hurt AC, Baas C, Deng YM, Roberts S, Kelso A, Barr IG, Performance of influenza rapid point-of-care tests in the detection of swine lineage A (H1N1) influenza viruses. Influenza Other Respir Viruses. 2009;3:171–6. 10.1111/j.1750-2659.2009.00086.x 19627374 PMC4634687

[7] Chan KH, Lai ST, Poon LL, Guan Y, Yuen KY, Peiris JS. Analytical sensitivity of rapid influenza antigen detection tests for swine-origin influenza virus (H1N1). J Clin Virol. 2009;45:205–7. 10.1016/j.jcv.2009.05.034 19539521

[8] Centers for Disease Control and Prevention. Updated interim recommendations for the use of antiviral medications in the treatment and prevention of influenza for the 2009–2010 seasons [cited 2010 Dec 4]. www.cdc.gov/h1n1/flu/ recommendations.htm#8

[9] Cooper NJ, Sutton AJ, Abrams KR, Wailoo A, Turner D, Nicholson KG, Effectiveness of neuraminidase inhibitors in treatment and prevention of influenza A and B: systematic review and meta-analysis of randomised control trials. BMJ. 2003;326:1235. 10.1136/bmj.326.7401.1235 12791735 PMC161558

[10] Aoki FY, MacLeod M, Paggiaro P, Carewicz O, El Sawy A, Wat C, Early administration of oral oseltamivir increases the benefits of influenza treatment. J Antimicrob Chemother. 2003;51:123–9. 10.1093/jac/dkg007 12493796

[11] Ling LM, Chow AL, Lye DC, Tan AS, Krishnan P, Cui L, Effects of early oseltamivir therapy on viral shedding in 2009 pandemic influenza A (H1N1) virus infection. Clin Infect Dis. 2010;50:963–9. 10.1086/651083 20180701

[12] Centers for Disease Control and Prevention. Emergency use authorization of Tamiflu®: fact sheet for health care providers [cited 2010 Dec 4]. http://www.cdc.gov/h1n1flu/eua/

[13] World Health Organization. Preliminary information important for understanding the evolving situation: novel influenza A (H2N1), briefing note 4, July 14, 2009 [cited 2010 Dec 21]. http://www.who.int/csr/diseae/swineflue/notes/h1n1_situation_20090724/en/index.html

[14] World Health Organization. Clinical management of human infection with pandemic (H1N1) 2009: revised guidance. November 2009 [cited 2010 Nov 1]. http://www.who.int/csr/resources/publications/swineflu/clinical_management/en/index.html

[15] Centers for Disease Control and Prevention. CDC issues interim recommendations for the use of influenza antiviral medications in the setting of oseltamivir resistance among circulating influenza A (H1N1) viruses, 2008–09 Influenza Season. Health Alert Network; December 19, 2008 [cited 2010 Nov 8]. http://www.bt.cdc.gov/HAN/han00279.asp

[16] Centers for Disease Control and Prevention (CDC). Oseltamivir-resistant 2009 pandemic influenza A (H1N1) virus infection in two summer campers receiving prophylaxis—North Carolina, 2009. MMWR Morb Mortal Wkly Rep. 2009;58:969–72. 19745803

[17] Dharan N, Gubareva LV, Meyer JJ, Okomo-Adhiambo M, McClinton RC, Marshall SA, Infections with oseltamivir-resistant influenza A (H1N1) virus in the United States. JAMA. 2009;301:1034–41. 10.1001/jama.2009.294 19255110

[18] US Food and Drug Administration. FDA and CDC information on potential “spot shortages” of supplies for treating and preventing novel influenza A (H1N1) [cited 2010 Nov 8]. http://www.fda.gov/oc/opacom/hottopics/H1N1flu/shortages.html

[19] Swerdlow DL, Finelli L, Bridges CB. 2009 H1N1 influenza pandemic: field and epidemiologic investigations in the United States at the start of the first pandemic of the 21st century. Clin Infect Dis. 2011;52(Suppl 1):S1–3. 10.1093/cid/ciq005 21342879

[20] Khandaker G, Dierig A, Rashid H, King C, Heron L, Booy R. Systematic review of clinical and epidemiological features of the pandemic influenza A (H1N1) 2009. Influenza Other Respir Viruses. 2011;5:148–56. 10.1111/j.1750-2659.2011.00199.x 21477133 PMC5657010

[21] ICD-9-CM Expert for Hospitals and Payers 2012, vols. 1, 2, and 3, 6th ed. Roseville (CA): Medicalcodingbooks.com Inc.; 2012.

[22] Navaratnam P, Jayawant SS, Pedersen CA, Balkrishnan R. Physician adherence to the national asthma prescribing guidelines: evidence from national outpatient survey data in the United States. Ann Allergy Asthma Immunol. 2008;100:216–21. 10.1016/S1081-1206(10)60445-0 18426140

[23] Cabana MD, Rand CS, Powe NR, Wu AW, Wilson MH, Abboud PA, Why don’t physicians follow clinical practice guidelines? A framework for improvement. JAMA. 1999;282:1458–65. 10.1001/jama.282.15.1458 10535437

[24] Chowell G, Bertozzi SM, Colchero MA, Lopez-Gatell H, Alpuche-Aranda C, Hernandez M, Severe respiratory disease concurrent with the circulation of H1N1 influenza. N Engl J Med. 2009;361:674–9. 10.1056/NEJMoa0904023 19564633

[25] Qian Y-H, Su J, Shi P, He E-Q, Shao J, Sun N, Attempted early detection of influenza A (H1N1) pandemic with surveillance data of influenza-like illness and unexplained pneumonia. Influenza Other Respir Viruses. 2011;5:e497–86. 10.1111/j.1750-2659.2011.00248.x PMC5780665 21668678

[26] Rothberg MB, Haessler SD, Brown RB. Complications of viral influenza. Am J Med. 2008;121:258–64. 10.1016/j.amjmed.2007.10.040 18374680 PMC7172971

[27] Centers for Disease Control and Prevention. Bacterial co-infections in lung tissue specimens from fatal cases of 2009 pandemic influenza A (H1N1)—United States, May–August 2009. MMWR Morb Mortal Wkly Rep. 2009;58:1071–4. 19798021

[28] Peltola VT, McCullers JA. Respiratory viruses predisposing to bacterial infections: role of neuraminidase. Pediatr Infect Dis J. 2004;23(Suppl):S87–97. 10.1097/01.inf.0000108197.81270.35 14730275

[29] Lee N, Cockram CS, Chan PK, Hui DS, Choi KW, Sung JJ. Antiviral treatment for patients hospitalized with severe influenza infection may affect clinical outcomes. Clin Infect Dis. 2008;46:1323–4. 10.1086/533477 18444878

[30] McGeer A, Green KA, Plevneshi A, Antiviral therapy and outcomes of influenza requiring hospitalization in Ontario, Canada. Clin Infect Dis. 2007;45:1568–75. 10.1086/523584 18190317

[31] Boivin G, Hardy I, Tellier G, Maziade J. Predicting influenza infections during epidemics with use of a clinical case definition. Clin Infect Dis. 2000;31:1166–9. 10.1086/317425 11073747

[32] Jayawardena S, Eisdorfer J, Indulkar S, Pal SA, Sooriabalan D, Cucco R. Prescription errors and the impact of computerized prescription order entry system in a community-based hospital. Am J Ther. 2007;14:336–40. 10.1097/01.mjt.0000209681.22077.b9 17667207

[33] Wasserfallen JB, Butschi AJ, Muff P, Biollaz J, Schaller MD, Pannatier A, Format of medical order sheet improves security of antibiotics prescription: the experiences of an intensive care unit. Crit Care Med. 2004;32:655–9. 10.1097/01.CCM.0000114835.97789.AB 15090943

[34] Kaushal R, Bates DW, Landrigan C, McKenna KJ, Clapp MD, Federico F, Medication errors and adverse drug events in pediatric inpatients. JAMA. 2001;285:2114–20. 10.1001/jama.285.16.2114 11311101

[35] Lesar TS, Lomaestro BM, Pohl H. Medication prescribing errors in a teaching hospital: a nine-year experience. Arch Intern Med. 1997;157:1569–76. 10.1001/archinte.1997.00440350075007 9236558

[36] Salomon L, Deray G, Jaudon MC, Chebassier C, Bossi P, Launay-Vacher V, Medication misuse in hospitalized patients with renal impairment. Int J Qual Health Care. 2003;15:331–5. 10.1093/intqhc/mzg046 12930048

[37] Cantú TG, Ellerbeck EF, Yun SW, Castine SD, Kornhauser DM. Drug prescribing for patients with changing renal function. Am J Hosp Pharm. 1992;49:2944–8. 1481798

[38] Raisch DW, Straight TM, Holodniy M. Thrombocytopenia from combination treatment with oseltamivir and probenicid: case report, MedWatch data summary, and review of the literature. Pharmacotherapy. 2009;29:988–92. 10.1592/phco.29.8.988 19637952

[39] Centers for Disease Control and Prevention. Neurologic complications associated with novel influenza A (H1N1) virus infection in children—Dallas, Texas, May 2009. MMWR Morb Mortal Wkly Rep. 2009;58:773–8. 19629027

[40] Fleming DM, Pannell RS, Elliot AJ, Cross KW. Respiratory illness associated with influenza and respiratory syncytial virus infection. Arch Dis Child. 2005;90:741–6. 10.1136/adc.2004.063461 15855178 PMC1720494

