# Supplementary material for: Evaluation of Diagnostic and Therapeutic Approaches for Suspected Influenza A(H1N1)pdm09 Infection, 2009–2010
Source: Emerg Infect Dis. 2012 Sep;18(9):1414–21. doi: 10.3201/eid1809.111564 (PMC3437695; doi:10.3201/eid1809.111564)
Supplement: Related texts

# Research article: Evaluation of Diagnostic and Therapeutic Approaches for Suspected Influenza A(H1N1)pdm09 Infection, 2009–2010

**DOI:** 10.3201/eid1809.111564

**Authors:** Vini Vijayan, Jennie Jing, Kenneth M. Zangwill

**Affiliations:** Los Angeles Biomedical Research Institute at Harbor–UCLA Medical Center, Torrance, California, USA

## Abstract

To assess adherence to real-time changes in guidelines for influenza diagnosis and use of oseltamivir during the 2009 influenza A(H1N1) pandemic, we reviewed medical records of patients with confirmed or suspected influenza-like illness (ILI) and those with no viral testing in a large Los Angeles (California, USA) hospital. Of 882 tested patients, 178 had results positive for influenza; 136 of the remaining patients received oseltamivir despite negative or no results. Oseltamivir use was consistent with national recommendations in >90%. Of inpatients, children were less likely than adults to have ILI at testing and to receive oseltamivir if ILI was found. Of outpatients, children were more likely to have positive test results; 20% tested did not have ILI or other influenza signs and symptoms. Twenty-five of 96 test-positive patients and 13 of 19 with lower respiratory tract disease were, inappropriately, not treated. Variations between practice and national recommendations could inform clinical education in future influenza seasons.

In April 2009, the novel influenza A(H1N1) pandemic influenza virus (influenza A[H1N1]pdm09) was identified as the cause of influenza outbreaks. Influenza disease caused by this strain rapidly spread, and in June 2009, the World Health Organization (WHO) declared a global pandemic. Disease activity peaked during May–June 2009, again in October 2009, and essentially disappeared by May 2010 (*1*–*3*). As with previous pandemics, the strain reemerged in the United States during the subsequent 2010–2011 influenza season and accounted for ≈25% of characterized strains (*4*).

During the pandemic, the Centers for Disease Control and Prevention (CDC) issued several guidances for healthcare providers for the identification and treatment of patients with suspected influenza A(H1N1)pdm09 disease (Figure 1). Several rapid influenza diagnostic tests for identification of the 2009 H1N1 strain were available, but their poor sensitivity soon became clear (*5*–*7*). CDC recommended that the neuraminidase inhibitor oseltamivir be used as a first-line treatment during the pandemic (*8*). Available data suggested that the drug was clinically effective, but only when given within <48 hours of symptom onset (*9*–*11*). These guidelines changed during the course of the pandemic as real-time epidemiologic, virologic, and clinical data emerged (*8*,*12*–*15*).

**Figure 1:** Centers for Disease Control and Prevention (CDC) guidance during the 2009 pandemic of influenza A(H1N1)pdm09 disease. LRTI, lower respiratory tract infection.

CDC initially recommended priority use of antiviral drugs for only hospitalized patients and those at increased risk for influenza-related complications. This recommendation reflected the knowledge that most persons infected with A(H1N1)pdm09 virus had self-limited, mild-to-moderate disease; that commercial and stockpiled supplies of oseltamivir were limited; and that the development of resistance was a concern, particularly since no other effective and easily administered antiviral drugs were available (*15*–*18*). Questions remained, however, with regard to the overall risks and benefits and appropriate dosage of the drug for very young and obese patients. In September 2009, CDC advised that rapid influenza diagnostic tests be prioritized for patients who were hospitalized or for whom a diagnosis of influenza could inform clinical decision making. Furthermore, CDC reinforced the idea that presumptive treatment should be administered to this group of patients and expanded the target group for treatment to include outpatients with risk factors for severe disease, even when test results were unknown (*5*). Clinical judgment was clearly a key factor in the clinical management of patients with possible A(H1N1)pdm09 disease.

Much has been published with regard to the epidemiology, virology, and clinical spectrum of A(H1N1)pdm09 illness (*19*,*20*), but no information is available with regard to diagnostic and therapeutic decision making of physicians or their adherence to national guidelines for ill patients. We conducted this study to evaluate the adherence of physicians to contemporaneous national guidelines for diagnosis and use of oseltamivir among patients with suspected or confirmed A(H1N1)pdm09 virus infection in the inpatient and outpatient settings.

## Methods

The study population included all persons who accessed care from May 1 to December 31, 2009, at Harbor–UCLA Medical Center (HUMC) in Los Angeles, California. HUMC is a 538-bed, urban, academic, teaching hospital; it serves a diverse population, which is ≈55% Latino, 11% Caucasian, 24% black, 4% Asian, and 4% Pacific Islander.

We conducted a retrospective cohort study to evaluate 3 issues: 1) adherence of clinicians to national recommendations for use of osteltamivir among patients with suspected or confirmed influenza virus infection; 2) appropriateness of patient selection for diagnostic testing; and 3) the likelihood of clinicians to prescribe antiviral drug therapy for persons with known influenza-like illness (ILI) or lower respiratory tract infection (LRTI), 2 conditions for which CDC specifically recommended antiviral drug therapy. For the first 2 objectives, we identified child and adult inpatients and those seen in the emergency department with A(H1N1)pdm09 disease by using 4 overlapping data sources, including the following: 1) prospectively collected electronic A(H1N1)pdm09 virus laboratory-based surveillance data obtained by the HUMC clinical virology laboratory and the Infection Prevention and Control Department; 2) electronic, pharmacy-based oseltamivir utilization data; and 3) data on point-of-care testing performed in the emergency department. These data were combined, and we reviewed the medical records of all patients with a positive laboratory test for influenza in the outpatient setting and of inpatients who had a laboratory test that was positive for influenza virus or were prescribed oseltamivir. Approval for human subjects research was obtained from the Los Angeles Biomedical Research Institute.

We performed a comprehensive review of medical records by using a standardized data collection instrument to identify demographic information and clinical characteristics of patients with the illness, including symptoms and signs and results of viral diagnostic testing and chest radiographs. Use of and indications for oseltamivir, including dose and duration of use, were recorded and, if oseltamivir was not prescribed, reasons for not using the drug were noted. We also recorded whether the patient exhibited risk factors for complications and death (from a preselected list that included concomitant cardiopulmonary, renal, liver, endocrine, blood, or metabolic disorders; immunosuppressive conditions; aspirin therapy; and neurologic conditions), diagnoses at admission or discharge, and length of stay.

We defined suspected influenza as illness in any patient for whom oseltamivir was prescribed by the treating clinician. We defined confirmed influenza disease as illness in a patient with a positive laboratory test result for the virus. To evaluate adherence to guidelines, we used the contemporaneous CDC definition for ILI (fever and cough with or without sore throat) and defined severe illness as requiring intensive care, a documented oxygen saturation of <92%, or both.

To assess the likelihood of clinicians to prescribe antiviral drug therapy for persons with known ILI or LRTI, we identified all inpatients and outpatients with possible upper or lower respiratory tract influenza disease by using International Classification of Diseases, Ninth Revision (ICD-9) diagnostic codes as follows: 079.89 (viral infection), 079.99 (viral infection not otherwise specified [NOS]), 460 (nasopharyngitis, acute), 462 (pharyngitis, acute), 465.8 (infectious upper respiratory, multiple sites, acute), 465.9 (infectious upper respiratory, multiple sites, acute NOS), 466.0 (bronchitis, acute), 466.19 (bronchiolitis, acute, due to other infectious organism), 478.9 (disease, upper respiratory /NOS), 480.1 (pneumonia caused by respiratory syncytial virus), 480.8 (pneumonia caused by virus), 480.9 (viral pneumonia unspecified), 484.8 (pneumonia in other infectious disease), 485 (bronchopneumonia, organism NOS), 486 (pneumonia, organism NOS), 487.0 (influenza with pneumonia), 487.1 (influenza with respiratory manifestation), 487.8 (influenza with manifestation), 488.1 (influenza caused by identified novel H1N1 influenza virus), 490 (bronchitis NOS), 780.6 (fever), 784.1 (pain, throat), 786.2 (cough) (*21*). The validity of the ICD-9–based ascertainment was assessed by using prospective emergency department triage ILI surveillance data collected beginning October 21, 2009, through the end of the study period.

From this group, we randomly selected 100 persons, stratified by age (50 persons <18 and 50 >18 years of age) by using SAS 9.2, Proc Samplesurvey (SAS Institute, Cary, NC, USA). Using medical record review, we then identified persons with ILI (defined above) or LRTI, defined by the presence of at least 1 specific lower respiratory tract sign, including tachypnea, retractions, or hypoxia (oxygen saturation <92%), and/or abnormal auscultatory findings (crackles/crepitations or wheezing), and/or unequivocal and abnormal radiographic findings.

We performed descriptive analyses of the above variables by using SAS version 9.2. Testing of proportions was performed by using χ^2^ or Fisher exact test as appropriate. All reported p values are 2-tailed and were considered significant if p<0.05.

## Results

### Entire Cohort

We identified 882 patients who were tested for influenza virus during the study period, among whom 178 (20%) tested positive. An additional 136 received oseltamivir but were not tested or had a negative laboratory test result for influenza virus. Overall, 232 (74%) of 314 patients had ILI, and 82 (26%) of 314 had a positive test result for influenza virus but did not meet the CDC-defined criteria for ILI. Of these 82, 36 (44%) had other signs or symptoms consistent with influenza, such as headache, myalgia, nausea, or diarrhea. We identified 218 (69%) inpatients among the 314 patients with confirmed or suspected influenza. Of those 314 patients, 55 (18%) were <2 years of age, 129 (41%) were 2–18 years of age, 89 (28%) were 19 to <50 years of age, 32 (10%) were 51 to <65 years of age, and 9 (3%) were >65 years of age. An underlying medical condition was recognized in 88 (48%) children (most commonly, asthma) and in 95 (52%) adults (most commonly, immunosuppression).

Oseltamivir was prescribed for 86 (66%) of 130 children and 89 (87%) of 102 adults with ILI. Oseltamivir was prescribed at the correct dosage and duration of therapy for 229 (95%) of 240 patients, and 216 (90%) of 240 patients received the drug <48 hours after symptom onset. Another 16 received the drug within 72 hours of disease onset. Severe illness was identified in 132 (42%) of 314 patients, 118 (89%) of whom received oseltamivir (Figure 2).

**Figure 2:** Total number of patients treated with oseltamivir by category, presence of influenza-like illness (ILI), and disease severity, Los Angeles, California, USA, 2009.

### Inpatients

Of 218 inpatients who received a diagnosis of or treatment for influenza, 107 (49%) were children, and 111 (51%) were adults. Laboratory testing was performed for 177 (81%) inpatients, and 74 (42%) were positive for influenza virus (Table). Oseltamivir was administered to 198 (91%) of 218 inpatients, among whom 110 (50%) had a negative test or no laboratory testing performed. Of the remaining 88 with a positive test result, 5 did not receive oseltamivir because the patient refused, the patient was “well appearing,” or patient’s onset of symptoms occurred >48 hours before they received a diagnosis.

**Table:** Patients who underwent testing or treatment for influenza by category, Los Angeles, California, USA, 2009*

Test results and treatment	Inpatients†	Outpatients‡
Influenza diagnostic test		
Patients tested for influenza		
Total	177/218 (81)	664/664 (100)
Adults	79/111 (71)	398/398 (100)
Children	98/107 (92)	266/266 (100)
Positive influenza test result		
Total	74/177 (42)	96/664 (14)
Adults	18/79 (23)	19/398 (5)
Children	56/98 (57)	77/266 (29)
ILI among patients with a positive test result		
Total	44/74 (59)	77/96 (80)
Adults	14/18 (78)	16/19 (84)
Children	30/56 (54)	61/77 (79)
Oseltamivir prescribed		
Patients with positive influenza test result	53/74 (72)	22/96 (23)
Patients with coexisting condition	145/155(94)	15/28 (54)
Patients with severe influenza disease	118/129 (91)	0/3 (0)
Median time from illness onset to treatment, d	2 (1–8)	2 (1–5)

*Values are no./total no. (%) unless otherwise indicated. ILI, influenza-like illness. †For inpatients who received a diagnostic test for influenza, N = 218; for outpatients who received a diagnostic test, N = 664. For inpatients who received oseltamivir, N = 218. In the outpatient setting, study cohort was identified through diagnostic testing only. Use of oseltamivir was evaluated only among those for whom a diagnostic test result was positive (N = 96).

Of the inpatients, we identified 68 (64%) of 107 children and 86 (77%) of 111 adults who had ILI at the time of laboratory testing (p<0.04). Oseltamivir was given to 58 (85%) of the 68 children with ILI and 84 (98%) of 86 adults with ILI (p<0.02). Oseltamivir was prescribed for 145 (94%) of 155 inpatients with an underlying medical condition and for 118 (91%) of 129 patients with severe illness.

The median interval from illness onset to initiation of antiviral treatment was 2 days (range 1–8). The dosage or duration of therapy, or both, was incorrect for 11 (5%) inpatients; for 6 inpatients, no adjustment was made for renal insufficiency. Of those 6 inpatients, 2 had chronic renal insufficiency after a transplant, 1 had diabetic nephropathy, and 3 had pneumonia and renal insufficiency. Three obese patients received a doubled dose of oseltamivir.

Receipt of the vaccine against influenza A(H1N1)pdm09 virus was documented in 61 (28%) of 218 patients, but 59 (97%) of them received the vaccine at hospital discharge. Only 1 patient had received the seasonal influenza vaccine before admission, and none received vaccine at discharge.

### Outpatients

We identified 664 patients who underwent rapid influenza diagnostic testing, of whom 77 (19%) of 398 children and 19 (7%) of 266 adults tested positive (p<0.001). Twenty percent of tests were carried out on patients without CDC-defined ILI and for whom no other indication was present. As noted in Figure 3, only 11% (73/664) of these tests were performed >2 weeks after CDC actively discouraged their use.

**Figure 3:** Rapid influenza diagnostic testing (RIDT) performed for outpatients with influenza-like illness (ILI), Los Angeles, California, USA, 2009.

Oseltamivir was prescribed for 37 (48%) of 77 outpatient children and 5 (26%) of 19 adults who tested positive for influenza (p>0.05), all at the appropriate dose and duration. As recommended, 35 (83%) of 42 received the drug <48 hours from symptom onset, and the remaining patients received the drug within 72 hours of symptom onset. Of 54 (56%) of 96 patients who tested positive and did not receive oseltamivir, 25 (46%) were not treated according to CDC guidelines, and 8 (15%) refused therapy. The reasons for not initiating oseltamivir therapy included onset of symptoms >48 hours previously and lack of an underlying medical condition. For 21 (39%) of the untreated patients, we found no documentation of the reason for withholding therapy.

We found 3 outpatients who had severe illness, none of whom received oseltamivir, and the reasons for withholding therapy could not be determined. Conversely, 16 (3%) of 522 patients with a negative test result received oseltamivir. The most common reasons documented for initiating therapy in this group included an underlying medical condition or concomitant diagnosis of pneumonia, ILI, or both, each consistent with CDC guidelines.

### Therapy for Patients with ILI or LRTI Not Tested for Influenza Virus

We reviewed records of 50 randomly selected outpatients with ICD-9 codes for ILI who were not tested for influenza virus. Only 3 patients (6%) received oseltamivir (as recommended by CDC). Of the remainder who did not receive the drug, the duration of illness was >48 hours, the patient was “well appearing,” or no underlying risk factors were found. The median time from illness onset to obtaining medical attention was 3.7 days (range 0–14 days); 22 (44%) sought treatment within 48 hours. Thirteen (26%) had an underlying medical condition (7 children and 6 adults). For each, however, there was an appropriate reason for withholding therapy, per CDC guidelines.

Among 50 outpatients with ILI and LRTI, 14 (28%) were admitted, 2 to the intensive care unit. The median time from illness onset to obtaining medical attention was 3 days (range 0–28 days); 31 (62%) of 50 sought treatment >48 hours after symptom onset. Eight of 25 (32%) children and 5 (20%) of 25 adults received oseltamivir, and 6 patients received the drug <48 hours from symptom onset. Oseltamivir was administered to 6 (38%) of 16 patients with severe illness and to 7 (25%) of 28 who had an underlying medical condition. The reason for not prescribing oseltamivir was documented in 5 charts, and the reasons included were that symptom onset was >48 hours from the visit to the hospital and that the patient was “well appearing.” Overall, 13 (68%) of 19 patients with LRTI who sought treatment within 48 hours of illness onset did not receive oseltamivir as recommended by CDC.

## Discussion

We believe that this study provides useful information with regard to the diagnostic and therapeutic behaviors of clinicians caring for patients with possible influenza virus infection. Although our data reflect physician behavior during the 2009–10 influenza A (H1N1) pandemic, the findings are likely applicable to any influenza year because diagnostic test performance, disease intensity, antiviral agent resistance, and virus strain affect clinical decision making each year.

We were interested in 2 general concepts: practice performance when influenza was clinically suspected and the potential for missed therapeutic opportunities when it was not. For the former, we found that providers’ practices were often consistent with CDC guidelines but notable deficiencies were also identified. In particular, a substantial proportion of potentially high-risk patients were not empirically treated, and a reason to withhold therapy could not be documented. This dynamic is similar to that for other medical conditions for which clinical practice guidelines are available: provider behavior at variance with the guideline may reflect available patient-level information or other immediate concerns (*22*,*23*). In any case, we have identified potential areas for targeted education of healthcare providers that should be supplemented by rapid dissemination and follow-up of national guidelines if and when they change over time.

We also found inconsistencies in the use of antiviral drug therapy, which was often at variance with contemporaneous guidelines. In our population, 25% of patients who received oseltamivir did not have ILI or another clear indication for treatment. During the pandemic, the drug was recommended for inpatients with ILI and outpatients with ILI and risk factors for severe illness if they had sought treatment within 48 hours of symptom onset (*5*). However, although too many outpatients without ILI received oseltamivir, too few (32%) received the drug despite having LRTI, a consistent indication for therapy. For most patients with LRTI, we could not identify a reasonable justification for withholding therapy. Not surprisingly, all of these patients received antibacterial agents, yet it remains unclear whether the clinicians actively considered influenza virus as a primary pathogen or risk factor for the presumed bacterial superinfection. Influenza virus infection and its association with secondary bacterial infection is well documented with influenza A(H1N1)pdm09 virus infection and with interepidemic disease (*24*–*27*). Treatment with antiviral drugs in this setting may lessen illness when superinfection exists (*26*,*28*). In this circumstance, greater recognition of the possibility of influenza virus infection and use of antiviral drug therapy may mitigate illness and lessen hospital costs (*29*,*30*).

We found that diagnostic practices were often inconsistent with contemporaneous guidelines. Nearly one third of patients were tested for influenza virus, despite the lack of ILI and ≈20% had no other indication for which testing might otherwise be justified (e.g., headache, myalgia). Previous work has shown that relatively few patients with influenza virus infection have systemic signs without fever, sore throat, or cough (*31*). Although changes in CDC recommendations were quickly disseminated to hospital clinicians by management memo, email, or face-to-face meetings, even more rapid communication and follow-up reminders may have enhanced adherence to guidelines.

We found that the dosage and duration of oseltamivir were generally consistent with CDC guidelines in ≈90% of all treated patients, and specifically for all outpatients. HUMC required the use of a preauthorization drug form that noted the appropriate age- and weight-based dose; an outpatient prescription for oseltamivir would not have been released without a completed form. Such tools have been shown to limit dosing errors (*32*,*33*). Also consistent with the CDC guidelines, >90% of hospitalized patients and patients with severe illness in our study received oseltamivir. Among outpatients, we noted that for ≈50%, an appropriate rationale for not providing oseltamivir was documented in the medical record.

Among the small number of dosing errors identified, >40% were related to inappropriate adjustment for renal insufficiency. More than 90% of oseltamivir is metabolized to oseltamivir carboxylate, 99% of which is eliminated by renal excretion, thus requiring dosage adjustment in this setting. Antimicrobial drug dosing errors are common (*34*,*35*), and a failure to adjust for renal impairment is a frequent underlying reason (*36*,*37*). Although controlled data are not available, oseltamivir has been associated with the development of thrombocytopenia, particularly when renal clearance is artificially lowered by concomitant administration of the drug probenicid (*38*). Attention should be given to patients’ renal function, particularly in the elderly (diminished renal clearance) and in those for whom higher doses may be recommended, such as the severely ill or obese (*8*).

We identified clinical management differences between how clinicians prescribed treatment for adult patients and how they prescribed treatment for children. Children who were inpatients were significantly less likely to have ILI at the time of testing and to receive treatment for ILI. When testing was carried out, children were also more likely to test positive for influenza virus than were adults, possibly because of the higher virus load in this population. These data also may reflect more overall testing of children, particularly young children who are more likely than adults to have nonspecific signs and symptoms (lethargy, poor feeding, abdominal pain) (*39*,*40*). In addition, infants and young children may not articulate symptoms of ILI (e.g., sore throat), leading to increased nonspecific testing and treatment of this population.

The main strength of this study is the comprehensive nature of case ascertainment, which included laboratory-based information and review of all prospectively collected logs for emergency department point-of-care testing. However, some patients who underwent testing for influenza virus may not have been noted in the outpatient log system. We appreciate that ICD-9 code data for ILI and LRTI may be nonspecific, but our prospectively collected ILI data (albeit for a limited portion of the surveillance period) validated the temporal trends for this diagnosis in the outpatient setting. We also did not include data from medical outpatient (nonemergency department) clinics where other patients with influenza may have been identified and treated, perhaps skewing our data to those who were more ill. As a retrospective study, our conclusions depend solely upon information documented in the medical record, which may be incomplete. Also, the use of an antiviral agent authorization form most likely improved the dosing practice, as has been shown in other settings (*32*,*33*). Last, our study population includes only a single academic medical center and therefore may not be representative of the region or the nation.

To our knowledge, similar studies of physician behavior with regard to influenza disease, and for A(H1N1)pdm09 disease in particular, have not been reported. We have identified variations in clinical practice in relation to national guidelines that suggest potential areas of education for future influenza seasons.

## References

[1] World Health Organization. New influenza A (H1N1) virus: global epidemiological situation June 2009. Wkly Epidemiol Rec. 2009;84:249–57.19537358

[2] Novel Swine-Origin Influenza A (H1N1) Virus Investigation Team; Dawood FS, Jain S, Finelli L, Shaw MW, Lindstrom S, et al. Emergence of a novel swine-origin influenza A (H1N1) virus in humans. N Engl J Med. 2009;360:2605–15. 10.1056/NEJMoa090381019423869

[3] Centers for Disease Control and Prevention. Hospitalized patients with novel influenza A (H1N1) virus infection—California, April–May, 2009. MMWR Morb Mortal Wkly Rep. 2009;58:536–41.19478723

[4] Centers for Disease Control and Prevention. Flu activity and surveillance, 2010 −2011 [cited 2011 Jun 5]. http://www.cdc.gov/flu/weekly/.

[5] Centers for Disease Control and Prevention. Interim guidance for the detection of novel influenza A virus using rapid influenza diagnostic tests [cited 2010 Dec 4]. http://www.cdc.gov/h1n1flu/guidance/rapid_testing.htm

[6] Hurt AC, Baas C, Deng YM, Roberts S, Kelso A, Barr IG, Performance of influenza rapid point-of-care tests in the detection of swine lineage A (H1N1) influenza viruses. Influenza Other Respir Viruses. 2009;3:171–6. 10.1111/j.1750-2659.2009.00086.x19627374PMC4634687

[7] Chan KH, Lai ST, Poon LL, Guan Y, Yuen KY, Peiris JS. Analytical sensitivity of rapid influenza antigen detection tests for swine-origin influenza virus (H1N1). J Clin Virol. 2009;45:205–7. 10.1016/j.jcv.2009.05.03419539521

[8] Centers for Disease Control and Prevention. Updated interim recommendations for the use of antiviral medications in the treatment and prevention of influenza for the 2009–2010 seasons [cited 2010 Dec 4]. www.cdc.gov/h1n1/flu/ recommendations.htm#8

[9] Cooper NJ, Sutton AJ, Abrams KR, Wailoo A, Turner D, Nicholson KG, Effectiveness of neuraminidase inhibitors in treatment and prevention of influenza A and B: systematic review and meta-analysis of randomised control trials. BMJ. 2003;326:1235. 10.1136/bmj.326.7401.123512791735PMC161558

[10] Aoki FY, MacLeod M, Paggiaro P, Carewicz O, El Sawy A, Wat C, Early administration of oral oseltamivir increases the benefits of influenza treatment. J Antimicrob Chemother. 2003;51:123–9. 10.1093/jac/dkg00712493796

[11] Ling LM, Chow AL, Lye DC, Tan AS, Krishnan P, Cui L, Effects of early oseltamivir therapy on viral shedding in 2009 pandemic influenza A (H1N1) virus infection. Clin Infect Dis. 2010;50:963–9. 10.1086/65108320180701

[12] Centers for Disease Control and Prevention. Emergency use authorization of Tamiflu®: fact sheet for health care providers [cited 2010 Dec 4]. http://www.cdc.gov/h1n1flu/eua/

[13] World Health Organization. Preliminary information important for understanding the evolving situation: novel influenza A (H2N1), briefing note 4, July 14, 2009 [cited 2010 Dec 21]. http://www.who.int/csr/diseae/swineflue/notes/h1n1_situation_20090724/en/index.html

[14] World Health Organization. Clinical management of human infection with pandemic (H1N1) 2009: revised guidance. November 2009 [cited 2010 Nov 1]. http://www.who.int/csr/resources/publications/swineflu/clinical_management/en/index.html

[15] Centers for Disease Control and Prevention. CDC issues interim recommendations for the use of influenza antiviral medications in the setting of oseltamivir resistance among circulating influenza A (H1N1) viruses, 2008–09 Influenza Season. Health Alert Network; December 19, 2008 [cited 2010 Nov 8]. http://www.bt.cdc.gov/HAN/han00279.asp

[16] Centers for Disease Control and Prevention (CDC). Oseltamivir-resistant 2009 pandemic influenza A (H1N1) virus infection in two summer campers receiving prophylaxis—North Carolina, 2009. MMWR Morb Mortal Wkly Rep. 2009;58:969–72.19745803

[17] Dharan N, Gubareva LV, Meyer JJ, Okomo-Adhiambo M, McClinton RC, Marshall SA, Infections with oseltamivir-resistant influenza A (H1N1) virus in the United States. JAMA. 2009;301:1034–41. 10.1001/jama.2009.29419255110

[18] US Food and Drug Administration. FDA and CDC information on potential “spot shortages” of supplies for treating and preventing novel influenza A (H1N1) [cited 2010 Nov 8]. http://www.fda.gov/oc/opacom/hottopics/H1N1flu/shortages.html

[19] Swerdlow DL, Finelli L, Bridges CB. 2009 H1N1 influenza pandemic: field and epidemiologic investigations in the United States at the start of the first pandemic of the 21st century. Clin Infect Dis. 2011;52(Suppl 1):S1–3. 10.1093/cid/ciq00521342879

[20] Khandaker G, Dierig A, Rashid H, King C, Heron L, Booy R. Systematic review of clinical and epidemiological features of the pandemic influenza A (H1N1) 2009. Influenza Other Respir Viruses. 2011;5:148–56. 10.1111/j.1750-2659.2011.00199.x21477133PMC5657010

[21] ICD-9-CM Expert for Hospitals and Payers 2012, vols. 1, 2, and 3, 6th ed. Roseville (CA): Medicalcodingbooks.com Inc.; 2012.

[22] Navaratnam P, Jayawant SS, Pedersen CA, Balkrishnan R. Physician adherence to the national asthma prescribing guidelines: evidence from national outpatient survey data in the United States. Ann Allergy Asthma Immunol. 2008;100:216–21. 10.1016/S1081-1206(10)60445-018426140

[23] Cabana MD, Rand CS, Powe NR, Wu AW, Wilson MH, Abboud PA, Why don’t physicians follow clinical practice guidelines? A framework for improvement. JAMA. 1999;282:1458–65. 10.1001/jama.282.15.145810535437

[24] Chowell G, Bertozzi SM, Colchero MA, Lopez-Gatell H, Alpuche-Aranda C, Hernandez M, Severe respiratory disease concurrent with the circulation of H1N1 influenza. N Engl J Med. 2009;361:674–9. 10.1056/NEJMoa090402319564633

[25] Qian Y-H, Su J, Shi P, He E-Q, Shao J, Sun N, Attempted early detection of influenza A (H1N1) pandemic with surveillance data of influenza-like illness and unexplained pneumonia. Influenza Other Respir Viruses. 2011;5:e497–86. 10.1111/j.1750-2659.2011.00248.xPMC578066521668678

[26] Rothberg MB, Haessler SD, Brown RB. Complications of viral influenza. Am J Med. 2008;121:258–64. 10.1016/j.amjmed.2007.10.04018374680PMC7172971

[27] Centers for Disease Control and Prevention. Bacterial co-infections in lung tissue specimens from fatal cases of 2009 pandemic influenza A (H1N1)—United States, May–August 2009. MMWR Morb Mortal Wkly Rep. 2009;58:1071–4.19798021

[28] Peltola VT, McCullers JA. Respiratory viruses predisposing to bacterial infections: role of neuraminidase. Pediatr Infect Dis J. 2004;23(Suppl):S87–97. 10.1097/01.inf.0000108197.81270.3514730275

[29] Lee N, Cockram CS, Chan PK, Hui DS, Choi KW, Sung JJ. Antiviral treatment for patients hospitalized with severe influenza infection may affect clinical outcomes. Clin Infect Dis. 2008;46:1323–4. 10.1086/53347718444878

[30] McGeer A, Green KA, Plevneshi A, Antiviral therapy and outcomes of influenza requiring hospitalization in Ontario, Canada. Clin Infect Dis. 2007;45:1568–75. 10.1086/52358418190317

[31] Boivin G, Hardy I, Tellier G, Maziade J. Predicting influenza infections during epidemics with use of a clinical case definition. Clin Infect Dis. 2000;31:1166–9. 10.1086/31742511073747

[32] Jayawardena S, Eisdorfer J, Indulkar S, Pal SA, Sooriabalan D, Cucco R. Prescription errors and the impact of computerized prescription order entry system in a community-based hospital. Am J Ther. 2007;14:336–40. 10.1097/01.mjt.0000209681.22077.b917667207

[33] Wasserfallen JB, Butschi AJ, Muff P, Biollaz J, Schaller MD, Pannatier A, Format of medical order sheet improves security of antibiotics prescription: the experiences of an intensive care unit. Crit Care Med. 2004;32:655–9. 10.1097/01.CCM.0000114835.97789.AB15090943

[34] Kaushal R, Bates DW, Landrigan C, McKenna KJ, Clapp MD, Federico F, Medication errors and adverse drug events in pediatric inpatients. JAMA. 2001;285:2114–20. 10.1001/jama.285.16.211411311101

[35] Lesar TS, Lomaestro BM, Pohl H. Medication prescribing errors in a teaching hospital: a nine-year experience. Arch Intern Med. 1997;157:1569–76. 10.1001/archinte.1997.004403500750079236558

[36] Salomon L, Deray G, Jaudon MC, Chebassier C, Bossi P, Launay-Vacher V, Medication misuse in hospitalized patients with renal impairment. Int J Qual Health Care. 2003;15:331–5. 10.1093/intqhc/mzg04612930048

[37] Cantú TG, Ellerbeck EF, Yun SW, Castine SD, Kornhauser DM. Drug prescribing for patients with changing renal function. Am J Hosp Pharm. 1992;49:2944–8.1481798

[38] Raisch DW, Straight TM, Holodniy M. Thrombocytopenia from combination treatment with oseltamivir and probenicid: case report, MedWatch data summary, and review of the literature. Pharmacotherapy. 2009;29:988–92. 10.1592/phco.29.8.98819637952

[39] Centers for Disease Control and Prevention. Neurologic complications associated with novel influenza A (H1N1) virus infection in children—Dallas, Texas, May 2009. MMWR Morb Mortal Wkly Rep. 2009;58:773–8.19629027

[40] Fleming DM, Pannell RS, Elliot AJ, Cross KW. Respiratory illness associated with influenza and respiratory syncytial virus infection. Arch Dis Child. 2005;90:741–6. 10.1136/adc.2004.06346115855178PMC1720494

# Research article: Evaluation of Diagnostic and Therapeutic Approaches for Suspected Influenza A(H1N1)pdm09 Infection, 2009–2010

**DOI:** 10.3201/eid1809.111564

**Authors:** Vini Vijayan, Jennie Jing, Kenneth M. Zangwill

**Affiliations:** Los Angeles Biomedical Research Institute at Harbor–UCLA Medical Center, Torrance, California, USA

## Medscape CME Quiz

## Earning CME Credit

To obtain credit, you should first read the journal article. After reading the article, you should be able to answer the following, related, multiple-choice questions. To complete the questions (with a minimum 70% passing score) and earn continuing medical education (CME) credit, please go to www.medscape.org/journal/eid. Credit cannot be obtained for tests completed on paper, although you may use the worksheet below to keep a record of your answers. You must be a registered user on Medscape.org. If you are not registered on Medscape.org, please click on the New Users: Free Registration link on the left hand side of the website to register. Only one answer is correct for each question. Once you successfully answer all post-test questions you will be able to view and/or print your certificate. For questions regarding the content of this activity, contact the accredited provider, CME@medscape.net. For technical assistance, contact CME@webmd.net. American Medical Association’s Physician’s Recognition Award (AMA PRA) credits are accepted in the US as evidence of participation in CME activities. For further information on this award, please refer to http://www.ama-assn.org/ama/pub/category/2922.html. The AMA has determined that physicians not licensed in the US who participate in this CME activity are eligible for *AMA PRA Category 1 Credits*™. Through agreements that the AMA has made with agencies in some countries, AMA PRA credit may be acceptable as evidence of participation in CME activities. If you are not licensed in the US, please complete the questions online, print the certificate and present it to your national medical association for review.

## Article Title: Evaluation of Diagnostic and Therapeutic Approaches for Suspected Influenza A(H1N1)pdm09 Infection, 2009–2010

## CME Questions

**1. You are seeing a 17-year-old young woman with a history of malaise, low-grade fever, sore throat, and frontal headache for 2 days. You suspect that this patient might have influenza. Based on the results of the current study, what should you consider regarding influenza testing?**

A. 99% of patients with a positive test for influenza met CDC-defined criteria for influenza-like illness (ILI)

B. Children had higher rates of positive testing as outpatients compared with adults

C. All tested patients had some indication suggesting influenza infection

D. The rates of positive testing exceeded 50% among both children and adults in the outpatient setting

**2. You decide to initiate treatment with oseltamivir as an outpatient. What does the current study demonstrate regarding the use of oseltamivir in the outpatient setting?**

A. Adults were more likely than children to receive oseltamivir

B. Nearly half of patients with a positive rapid influenza diagnostic test (RIDT) were not treated according to CDC guidelines

C. Half of patients receiving oseltamivir had a negative RIDT

D. One quarter of patients received oseltamivir within 48 hours of the onset of symptoms

**3. The patient returns to your clinic 2 days later with worsening cough, dyspnea, and fever. You decide to admit her to the hospital. What does the current study demonstrate regarding the management of influenza in the inpatient setting?**

A. Over 90% of inpatients received oseltamivir

B. Over 90% of inpatients who received oseltamivir had positive influenza testing

C. Most patients prescribed oseltamivir received an incorrect dose or duration of therapy

D. The most common error in prescribing oseltamivir was extending the duration of therapy

**4. What else should you consider regarding the management of influenza in the setting of lower respiratory tract infection (LRTI) in the current study?**

A. All patients with cases of LRTI were admitted to the hospital

B. Most patients presented within 2 days of symptom onset 

C. Two thirds of patients failed to receive appropriate treatment

D. The principal reason that patients failed to receive oseltamivir was the belief of physicians that the drug would not work

## Activity Evaluation

**Table:** 

**1. The activity supported the learning objectives.**				
Strongly Disagree				Strongly Agree
1	2	3	4	5
**2. The material was organized clearly for learning to occur.**				
Strongly Disagree				Strongly Agree
1	2	3	4	5
**3. The content learned from this activity will impact my practice.**				
Strongly Disagree				Strongly Agree
1	2	3	4	5
**4. The activity was presented objectively and free of commercial bias.**				
Strongly Disagree				Strongly Agree
1	2	3	4	5
